# Effects of four cooking methods on flavor and sensory characteristics of scallop muscle

**DOI:** 10.3389/fnut.2022.1022156

**Published:** 2022-10-12

**Authors:** Yueyao Wang, Guifang Tian, Kemin Mao, Bimal Chitrakar, Zhongxuan Wang, Jie Liu, Xinzhong Bai, Yaxin Sang, Jie Gao

**Affiliations:** ^1^College of Food Science and Technology, Hebei Agricultural University, Baoding, China; ^2^Shandong Longsheng Food Co., Ltd., Laoling, China

**Keywords:** scallop, volatile flavor compounds, free amino acid, HS-GC-IMS, electronic nose, electronic tongue

## Abstract

This work aimed to explore the influence of four different cooking methods (Boiling, roasting, frying, and microwaving) on the sensory characteristics of scallop muscles. Headspace-gas chromatography-ion mobility spectrometry (HS-GC-IMS) and electronic nose (e-nose) were combined to analyze the aroma of scallops. Combined with the results of free amino acids and electronic tongue (e-tongue), the taste changes of different samples were analyzed. Furthermore, texture profile and microstructure analysis jointly showed the influence of cooking methods on texture. The results showed that frying was the most suitable cooking method for scallop muscle because it resulted the best tasted products, boiled scallops retain the highest similarity to fresh scallops. Besides, a higher level of lipid oxidation and Maillard reaction resulted in significant increase in aldehydes, ketones, furans, umami, and sweet amino acid. For the boiled sample, the loss of water-soluble compounds and less fat oxidation resulted in fewer flavor substances and free amino acids, along with looser organizational structure and poorer sensory quality. The research showed that besides the texture of scallop muscle, volatile organic compounds and free amino acids as well as their mutual roles in taste and smell were also important to sensory receptivity.

## Introduction

Scallop is a major cultivated shellfish in China and its the annual production output ranks top in the world. More than 90% was produced in China (1). Scallop varieties, namely *Argopecten irradians*, *Patinopecten yessoensis*, and *Chlamys farreri* are the three major scallops produced in the coastal areas of China (2). Among them, *Argopecten irradians* are mainly cultivated in northern coastal areas belonging to Shandong and Liaoning province. As a common kind of shellfish, *Argopecten irradians* are widely produced and consumed because of their short growth cycle and fast growth speed (3). However, fresh scallops easily deteriorate in a short time and generate an off-flavor, which limits their long-distance transport and storage life. Cooking can make the scallop safe to consume by killing pathogens; at the same time, its flavor is improved to a various degree. In recent years, there have been many studies on the effects of different cooking methods on the flavor, taste and sensory properties of aquatic products. Chen et al. (4) compared tilapia muscles heated through microwaving, roasting, steaming, and boiling and found that the four heating methods had significantly different influence on the flavor. Besides, due to varying heating principles, heating temperatures and other factors, different heating methods affect food organization and structure, which ultimately affect the food taste by changing muscle fiber structure and water contents. Lee et al. (5) found that palatability of white-striping chicken breasts changed after heating and the meat roasted in an oven tasted harder and chewier than that cooked by sous-vide. At present, the research on scallop mainly focuses on the influence of drying method on its flavor and taste substances (6, 7); however, there is little research on its cooking method. Therefore, the changes in flavor and sensory characteristics due to different cooking methods may be an interesting area in the sensory study of scallops.

However, the way to assess the flavor of heated products is as important as heating methods. HS-GC-IMS is an emerging flavor analytical instrument. Being highly sensitive, it can not only qualitatively and quantitatively analyze volatile organic compounds but also compare different samples of flavor substances more intuitively. E-nose and e-tongue are the bionic systems that simulate human senses of smell and taste; however, they have weaknesses as well. For example, they can’t identify concrete substances; so, they can’t fully replace the analytical instruments or sensory analysis (8). In recent years, the combined use of multiple instruments has become a popular trend, as it can provide more comprehensive and diversified information for the research on food flavor and taste characteristics (9). Established literature combined HS-GC-IMS, e-nose, e-tongue and amino acid detection to represent the flavor and taste changes of salmonid. The results showed that multiple instruments can complement each other in validating analysis results, thereby differentiating samples in a more comprehensive and effective manner (10). However, no report about the application of the combined use of these instruments to scallops have been available so far.

This work combined HS-GC-IMS and e-nose to analyze the aroma of scallop muscle after various cooking methods, including boiled in water, roasted, fried and heated by microwave. Combining the free amino acids in scallops and e-tongue results, we analyzed the taste changes of scallop muscle heated in different ways. Through sensory assessment, microstructure observation and texture profile analysis, we analyzed the sensory characteristics of heated scallop muscle. Finally, we analyzed how sensory assessment was correlated to taste and aroma. The research results can provide a basis for choosing the heating method suitable for scallop muscle for better sensory properties.

## Materials and methods

### Materials

Fresh scallops (*Argopectens irradias*) and corn oil were purchased from a local supermarket in Baoding, Hebei, China. Chemicals, such as sulfosalicylic acid, sodium citrate and ninhydrin were obtained from Sinopharm Chemical Reagent Co., Ltd. (Shanghai, China). The mixed amino acid standard solution (HPLC grade, amino acids in this standard were 2.5 μmol per mL in 0.1 N HCl, except L-cystine at 1.25 μmol per mL) was purchased from Sigma-Aldrich Chemical Co. (St. Louis, MO, USA).

### Treatment of samples

Fresh scallops were cleaned and shell-removed to get muscle parts; they were divided into five groups. The scallop without cooking was the control sample (CK). For boiling cooking, the sample was heated in boiling water for 10 min (BS). The roasted scallops (RS) were prepared by dry roasting inside a preheated oven (200°C) for 10 min; the samples were turned over at the fifth min. Scallops were fried in a pan containing preheated corn oil (150°C) for 6 min (FS). Microwaved scallops (MS) were obtained by heating them in a microwave oven at 400 W for 2 min. After cooling, all the samples were packed in zip-lock aluminum bags and kept in a fridge until further analysis.

### Volatile compounds analysis by headspace-gas chromatography-ion mobility spectrometry

Headspace-gas chromatography-ion mobility spectrometry (HS-GC-IMS) (FlavourSpec^®^, G.A.S., Dortmund, Germany) was used to analyze the volatile compounds of scallop muscle following the method of Li et al. (11) with slight modification. Before analysis, 2 g of the sample was taken into a 20 mL glass bottle and then incubated with swirling at 500 r/min for 10 min at 80°C. Then, a syringe heated to 85°C was used to inject 1.0 mL of headspace gas. The chromatographic column used was MXT-5 (15 m × 0.53 mm i.d., 1°μm film thickness; Restek Corporation, Bellefonte, PA, USA). Nitrogen (99.99% purity) was used as the carrier gas.

The elution program was as follows: 2 mL/min for 2 min; 10 mL/min within 8 min; 100 mL/min within 10 min; and 150 mL/min within 20 min. At 45°C, the substance was ionized and further separated in the IMS ionization chamber. During analysis, C4-C9 n-ketones (Sinopharm Chemical Reagent Co., Ltd., Beijing, China) was used as reference to identify the retention index (RI) of the substance. Then, RI and drift time (DT) were compared with GC × IMS Library.

### Free amino acids analysis by automatic amino acid analyzer

The method of Zhang et al. (12) was used to analyze free amino acids by using an amino acid analyzer (Biochrom 30+, UK) with slight modification. An accurately weighed (2 g) ground sample was dissolved in 10 mL of water and kept for 24 h. The supernatant was mixed with sulfosalicylic acid (5%, v/v). The mixture was centrifuged at 6,000 × g for 10 min and the supernatant was dried in a rotary evaporator; the residue was dissolved in 1 mL sodium citrate buffer and then filtered through 0.45 μm filter for the detection free amino acids at 570 nm (440 nm was used for proline detection). Standard curves were prepared by using external standards. Altogether, 17 free amino acids considered were: glycine (Gly), alanine (Ala), arginine (Arg), glutamic acid (Glu), cysteine (Cys), tyrosine (Tyr), methionine (Met), lysine (Lys), aspartic acid (Asp), proline (Pro), threonine (Thr), isoleucine (Ile), leucine (Leu), histidine (His), phenylalanine (Phe), valine (Val), and serine (Ser).

### Electronic nose analysis

The PEN-3 electronic nose (Airsense Technology Co., Ltd., Germany) was used to differentiate the flavor of raw scallop muscle from that of the scallop muscles cooked in four different ways. The e-nose had 10 metal receptors in total. Each receptor was sensitive to a specific type of substances. Sample (2.0 g) was cut into 2 mm × 2 mm pieces and put into a 20 mL glass bottle; after incubation at 60°C for 10 min, testing was done for 120 s. The e-nose was cleaned before each testing.

### Electronic tongue analysis

The SA-4028 electronic tongue (Ensoul, Beijing, China) was used to differentiate the taste of raw scallop muscle from cooked samples. Accurately weighed (10 g) cut sample was mixed with deionized water at 1:8 ratio and homogenized (Supor, Hangzhou, China) for 2 min at 32,000 r/min until well mixed. Supernatant was collected after centrifugation at 10,000 × g for 10 min, which was used for e-tongue analysis. The electronic tongue used sensors to detect soluble substances in the liquid sample to generate a signal response value of each sensor for analysis (13).

### Sensory evaluation analysis

Ten trained tasters (1:1 male: female ratio; aged between 20 and 25) were selected to assess sensory characteristics. Each taster had an independent space and was not disturbed by another taster. The scallop muscle sample was presented in a clean and transparent cup with a random 3-digit code. After each tasting, the panelist was normalized their mouth by a bite of biscuit and then cleaned with water. Scores were given for odor, taste, texture, and appearance; the average score from all the panelists was considered for the data analysis.

### Texture profile and microstructure analysis

Using TMS-Pro texture profile analyzer (FTC, USA) with a cylindrical probe (50 mm dia.), texture parameters were analyzed (14). The measurement speed was 1 mm/s with a deformation of 30%; two consecutive compressions were made within a 5 s interval time. For microstructure analysis, samples were cut into cubes (5 mm × 5 mm × 1 mm) and fixed on the sample support using a double-sided adhesive tape. The sample was viewed through scanning electron microscope (SU8010, Hitachi, Japan) at 500× magnification after coating with gold under vacuum (15).

### Statistical analysis

Correlation analysis of the differences among the samples was conducted using the SPSS 23.0 software (IBM, Armonk, NY, USA). The significance of the differences was conducted with Duncan multiple comparison method, considering *P* < 0.05 as the significant difference. Origin 2021 was used to conduct radar map-based visual analysis of the samples. Advanced Heatmap Plots was performed using the OmicStudio tools from https://www.omicstudio.cn. Main component analysis was performed using https://www.chiplot.online/. Correlation analysis and graphic presentations were generated using the R “corrplot” package (16). For correlation network diagram, cytoscape (Version 3.9.1) was used.

## Results and discussion

### Analysis of volatile flavor compounds in scallop samples

**FIGURE 1:**
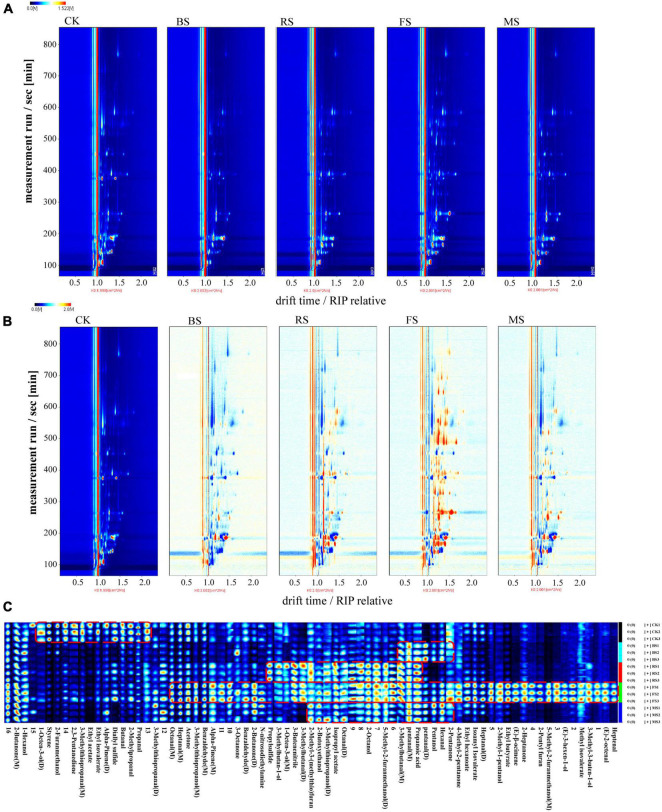
Headspace-gas chromatography-ion mobility spectrometry (HS-GC-IMS) topographic plots and gallery plot of five treatment groups. **(A)** Topographic plot of HS-GC-IMS spectra; **(B)** Comparison of spectrogram results. **(C)** Gallery plot of five treatment groups. (M) and (D) denote monomer and dimer, respectively (CK, the control group; BS, boiled sample; RS, oven heated sample; FS, fried sample; MS, microwaved sample).

Headspace-gas chromatography-ion mobility spectrometry was used to identify the volatile organic compounds in scallop muscle and analyze the change in flavor components among different samples. Figure 1A was the HS-GC-IMS topographic plot of the five samples. Each point on the left and right sides of RIP peak (reactive ion peak) represented a volatile flavor substance. The darker the red was, the higher the content was. It can be seen that the flavor components in the sample are well separated. Some substances disappeared and others appeared in different cooking steps. Figure 1B showed the comparison of the topographic plots of the five samples of scallops, through which we can observe the changes caused by the four heating methods to the flavor substances of scallops more clearly. The flavor substance topographic plot of raw scallop muscle was used as the reference. The red spots indicated an increased concentration of flavor substances, while the blue spots indicated their decreased concentration. These four cooking methods showed the changes in flavor substances in various degrees; the highest increase was observed in the fried scallop, while the lowest increase was found in the boiled ones.

By testing and analyzing through HS-GC-IMS (Table 1), the volatile organic compounds in raw scallop muscle and the scallop muscle cooked in different ways were 48 in number; the varieties included 12 aldehydes, 11 alcohols, 7 ketones, 7 esters, 1 acid, and 10 others; aldehydes and alcohols were the major compounds. Most aldehydes and ketones showed stronger response signals in the fried samples. The HS-GC-IMS gallery plot of the volatile flavor compounds in different samples were shown in Figure 1C, in which, each row represented the response signals of the volatile flavor compounds in one sample, and each column represented the response signals of each volatile flavor compound in different samples. The gallery plot enabled more intuitive comparison between the volatile flavor compounds in scallop samples. The categories of flavor substances in all samples were similar but the contents were different. Different cooking methods resulted different characteristic flavors, among which, the FS was loaded with a greater variety of volatile compounds with higher contents, while the result was opposite for the BS. The increase of such compounds in RS and MS samples was similar in terms of flavor areas. Except the BS samples, the contents of aldehydes and ketones increased after heating, particularly in case of fried samples, which was indicated by more red spots in the HS-GC-IMS topographic plots, ultimately contributing to rich flavors. The change might be resulted from the fact that the fried scallops absorbed unsaturated fatty acid from the oil during frying, which caused more lipid oxidation in the FS samples; the process was accelerated by the higher frying temperature (17). The lower contents of ketones and aldehydes in the BS samples was probably related to the lower heating temperature boiling water. During boiling, some oil and hydrolysis of scallops fat might get hydrolyzed into fatty acids and dissolved in water, causing the loss of flavor substances (18).

**TABLE 1:** Contents of volatile compounds in scallops identified by HS-GC-IMS.

S.No	Volatile compounds	CAS#	Formula	Retention index	Retention time (sec)	Drift time (a.u.)	Peak volume (a.u)				
							Raw	Boiled	Roasted	Fried	Microwaved
	**Aldehydes**										
1	Octanal (M)	C124130	C8H16O	998.6	585.19	1.40355	1560.84 ± 189.75^bc^	1,149.96 ± 89.48^c^	1,591.63 ± 220.91^bc^	2,338.03 ± 158.42^a^	1,658.26 ± 471.04^b^
2	Octanal (D)	C124130	C8H16O	980.3	551.031	1.41421	232.85 ± 28.60^b^	324.16 ± 42.80^ab^	457.44 ± 34.30^a^	460.66 ± 49.02^a^	405.18 ± 158.58^a^
3	Butanal	C123728	C4H8O	606.4	148.651	1.1028	1,684.24 ± 337.55^a^	835.52 ± 243.06^b^	608.64 ± 159.10^b^	696.62 ± 132.22^b^	483.23 ± 97.56^b^
4	Heptanal (M)	C111717	C7H14O	893.4	390.536	1.32983	1,810.60 ± 204.96^b^	1,090.96 ± 98.65^c^	1,637.35 ± 79.79^b^	2,236.26 ± 141.40^a^	1,565.95 ± 427.46^b^
5	Heptanal (D)	C111717	C7H14O	891.2	387.193	1.69606	580.77 ± 136.37^b^	212.80 ± 43.22^c^	392.64 ± 61.97^bc^	987.33 ± 165.29^a^	443.71 ± 234.14^bc^
6	Hexanal	C66251	C6H12O	765.5	247.461	1.25116	969.34 ± 79.94^b^	1,719.31 ± 202.63^a^	583.14 ± 176.96^b^	1,895.02 ± 113.63^a^	2,131.80 ± 482.75^a^
7	Pentanal (M)	C110623	C5H10O	685.1	182.891	1.18647	338.24 ± 15.51^c^	1,220.24 ± 129.07^a^	915.73 ± 126.91^b^	915.02 ± 94.67^b^	1,225.22 ± 67.69^a^
8	Pentanal (D)	C110623	C5H10O	688.8	184.604	1.42917	534.70 ± 76.20^d^	4,202.00 ± 638.70^a^	1,380.45 ± 414.08^c^	2,412.52 ± 188.12^b^	2,787.59 ± 618.32^b^
9	2-Methylpropanal	C78842	C4H8O	591.1	142	1.2895	1,292.00 ± 200.15^a^	380.77 ± 43.57^c^	499.28 ± 159.83^b^	878.90 ± 292.51^b^	387.50 ± 126.03^c^
10	Propanal	C123386	C3H6O	501	102.771	1.05695	4,461.92 ± 605.51^a^	2,752.13 ± 237.34^b^	2,617.10 ± 83.29^b^	2,920.20 ± 407.09^b^	2,330.30 ± 123.09^b^
11	3-Methylthiopropanal (D)	C3268493	C4H8OS	897.3	397.733	1.08839	272.26 ± 156.74	269.34 ± 140.22	262.81 ± 81.65	433.24 ± 179.28	539.24 ± 158.28
12	3-Methylthiopropanal (M)	C3268493	C4H8OS	909.9	421.061	1.0907	646.15 ± 67.5^b^	245.65 ± 48.67^c^	402.43 ± 66.15^c^	1,051.41 ± 183.06^a^	673.04 ± 198.11^b^
13	3-Methylbutanal (D)	C590863	C5H10O	647.9	166.712	1.40053	171.15 ± 48.04^b^	343.78 ± 147.69^b^	1,042.69 ± 340.18^a^	1,246.63 ± 121.38^a^	148.23 ± 63.22^b^
14	3-Methylbutanal (M)	C590863	C5H10O	643.3	164.716	1.17038	1,293.70 ± 155.10^c^	1,697.49 ± 281.11^b^	2,581.91 ± 232.83^a^	2,598.72 ± 56.93^a^	1,197.87 ± 139.95^c^
15	Benzaldehyde (D)	C100527	C7H6O	962.3	517.846	1.46872	194.27 ± 23.70^a^	108.77 ± 70.00^b^	115.89 ± 21.79^b^	221.67 ± 61.04^a^	170.39 ± 59.60^ab^
16	Benzaldehyde (M)	C100527	C7H6O	961.1	515.695	1.14858	1,241.38 ± 131.71^a^	656.23 ± 132.56^b^	1,050.69 ± 125.63^a^	1,379.00 ± 191.09^a^	1,101.30 ± 321.41^a^
17	(E)-2-octenal	C2548870	C8H14O	1, 054.1	693.07	1.33622	61.48 ± 15.73^b^	58.99 ± 14.06^b^	54.80 ± 21.18^b^	177.81 ± 28.53^a^	70.34 ± 23.48^b^
18	Heptenal	C18829555	C7H12O	946.5	488.68	1.66426	79.79 ± 8.77^b^	85.94 ± 28.43^b^	86.67 ± 14.32^b^	321.66 ± 73.04^a^	74.11 ± 5.01^b^
	**Ketones**										
19	2,3-Pentanedione	C600146	C5H8O2	694.7	189.449	1.30753	1732.37 ± 498.37^a^	494.01 ± 55.05^b^	432.95 ± 71.98^b^	789.69 ± 262.76^b^	466.76 ± 53.94^b^
20	2-Butanone (M)	C78933	C4H8O	582.7	138.338	1.06914	1,499.15 ± 187.65^b^	1,242.16 ± 144.04^b^	1,432.49 ± 244.06^b^	1,905.86 ± 42.34^a^	1,962.82 ± 240.26^a^
21	2-Butanone (D)	C78933	C4H8O	586	139.77	1.24463	947.80 ± 141.81^c^	434.01 ± 148.43^c^	907.54 ± 325.54^c^	2,385.43 ± 267.10^a^	1,735.36 ± 594.31^b^
22	Acetone	C67641	C3H6O	509.5	106.491	1.12538	4,966.29 ± 728.31^ab^	1,825.95 ± 692.47^d^	3,339.89 ± 658.00^c^	5,492.55 ± 788.33^a^	3,790.89 ± 996.56^bc^
23	2-Pentanone	C107879	C5H10O	691.8	187.072	1.3674	427.62 ± 32.96^b^	475.25 ± 97.95^ab^	348.00 ± 41.85^b^	624.80 ± 147.93^a^	418.66 ± 66.63^b^
24	4-Methyl-2-pentanone	C108101	C6H12O	718.5	208.908	1.17828	127.92 ± 28.52^c^	212.12 ± 20.45^b^	222.42 ± 44.79^b^	453.20 ± 35.22^a^	201.42 ± 8.58^b^
25	2-Heptanone	C110430	C7H14O	883.4	378.117	1.25837	193.63 ± 33.67^b^	160.24 ± 31.00^b^	204.95 ± 29.57^b^	473.34 ± 11.90^a^	178.63 ± 27.94^b^
26	3-Octanone	C106683	C8H16O	979	548.637	1.29835	230.43 ± 87.28	383.83 ± 221.98	247.59 ± 33.49	353.25 ± 200.17	326.75 ± 138.64
	**Alcohols**										
27	1-Octen-3-ol (M)	C3391864	C8H16O	1, 005.1	597.833	1.16261	268.00 ± 82.05^bc^	116.62 ± 36.97^c^	422.15 ± 146.50^a^	308.75 ± 23.25^ab^	148.50 ± 45.34^c^
28	1-Octen-3-ol (D)	C3391864	C8H16O	981.2	552.815	1.5881	376.05 ± 67.13^a^	134.96 ± 26.77^b^	140.26 ± 9.65^b^	189.29 ± 20.11^b^	142.10 ± 24.14^b^
29	2-Furanmethanol	C98000	C5H6O2	879.6	373.738	1.11127	2,126.07 ± 508.77^a^	940.06 ± 477.91^b^	559.21 ± 59.22^b^	612.04 ± 72.76^b^	428.35 ± 128.47^b^
30	Pentanol	C71410	C5H12O	762.5	245.031	1.5084	272.27 ± 53.88^bc^	561.85 ± 86.42^ab^	104.90 ± 21.23^c^	635.18 ± 95.36^a^	797.66 ± 362.90^a^
31	1-Hexanol	C111273	C6H14O	868.5	360.73	1.31603	215.32 ± 52.28	251.55 ± 26.40	186.63 ± 60.89	233.42 ± 28.26	218.71 ± 10.15
32	5-Methyl-2-furanmethanol (D)	C3857258	C6H8O2	962.2	517.639	1.27804	125.71 ± 8.02^b^	117.75 ± 25.06^b^	281.24 ± 73.18^a^	307.69 ± 20.07^a^	247.10 ± 24.81^a^
33	5-Methyl-2-furanmethanol (M)	C3857258	C6H8O2	947.6	490.757	1.25894	141.27 ± 32.12^b^	96.65 ± 5.06^b^	101.41 ± 5.06^b^	1,273.85 ± 163.04^a^	128.85 ± 9.19^b^
34	2-Methyl-1-pentanol	C105306	C6H14O	838.4	325.853	1.29886	65.51 ± 3.96^c^	60.15 ± 2.30^c^	61.27 ± 3.56^c^	204.06 ± 4.68^a^	75.80 ± 6.04^b^
35	3-Methylthiopropanol (D)	C505102	C4H10OS	979.6	549.783	1.45532	101.01 ± 6.51	113.81 ± 41.62	172.54 ± 26.15	139.22 ± 44.61	147.65 ± 69.63
36	3-Methylthiopropanol (M)	C505102	C4H10OS	977.9	546.755	1.10208	3,943.78 ± 830.80^a^	753.65 ± 62.89^b^	1,009.20 ± 190.33^b^	1,409.18 ± 585.99^b^	1,008.06 ± 267.41^b^
37	(E)-3-Hexen-1-ol	C928972	C6H12O	840.3	327.949	1.51447	46.73 ± 4.22^b^	46.89 ± 5.42^b^	46.43 ± 11.29^b^	139.90 ± 9.70^a^	41.03 ± 2.47^b^
38	3-Methyl-3-buten-1-ol	C763326	C5H10O	718.9	209.301	1.29057	53.01 ± 4.34^c^	63.28 ± 6.17^c^	81.48 ± 14.34^b^	183.96 ± 8.32^a^	54.35 ± 7.62^c^
39	3-Methylbutan-1-ol	C123513	C5H12O	728.2	216.879	1.23522	245.47 ± 49.00^b^	166.00 ± 17.49^b^	332.23 ± 57.31^a^	226.05 ± 20.41^b^	193.90 ± 64.42^b^
40	2-Octanol	C123966	C8H18O	998.1	584.168	1.45925	279.90 ± 49.86^d^	353.53 ± 14.36^c^	727.53 ± 7.01^a^	772.08 ± 53.17^a^	603.05 ± 33.20^b^
	**Esters**										
41	Isoamyl isovalerate	C659701	C10H20O2	1, 092	766.687	1.47268	959.87 ± 191.18^b^	496.31 ± 35.18^d^	674.76 ± 123.03^cd^	1,457.78 ± 130.53^a^	818.84 ± 193.30^bc^
42	Ethyl hexanoate	C123660	C8H16O2	996.6	581.156	1.81994	256.86 ± 61.17^b^	158.51 ± 17.83^b^	244.71 ± 64.05^b^	520.51 ± 93.99^a^	274.56 ± 110.95^b^
43	Ethyl isovalerate	C108645	C7H14O2	935	467.501	1.26531	432.74 ± 161.90^a^	177.01 ± 31.59^b^	208.06 ± 37.82^b^	262.17 ± 63.22^b^	192.50 ± 31.34^b^
44	Ethyl butyrate	C105544	C6H12O2	786.9	265.993	1.56437	681.82 ± 213.13^b^	572.15 ± 63.23^b^	696.44 ± 169.79^b^	4,443.93 ± 275.36^a^	870.54 ± 339.52^b^
45	Ethyl acetate	C141786	C4H8O2	601.7	146.601	1.33466	1,175.58 ± 580.67^a^	220.51 ± 83.34^b^	205.97 ± 114.91^b^	247.50 ± 125.96^b^	100.61 ± 25.34^b^
46	Isopropyl acetate	C108214	C5H10O2	591.3	142.074	1.15412	529.36 ± 34.19^c^	632.95 ± 125.79^c^	1,534.86 ± 353.64^a^	1,159.58 ± 40.41^b^	1,073.59 ± 16.61^b^
47	Methyl isovalerate	C556241	C6H12O2	777.8	257.532	1.19206	127.50 ± 25.58^b^	144.16 ± 9.51^b^	130.68 ± 11.36^b^	198.83 ± 5.29^a^	136.10 ± 6.00^b^
	**Acids**										
48	Propanoic acid	C79094	C3H6O2	691.9	187.137	1.26579	617.76 ± 72.72^c^	1,349.68 ± 161.62^b^	1,563.14 ± 64.98^a^	1,461.05 ± 51.72^ab^	1,383.61 ± 89.50^ab^
	**Others**										
49	Alpha-pinene (D)	C80568	C10H16	927	452.716	1.22059	736.52 ± 415.78	354.36 ± 53.59	439.73 ± 98.28	476.24 ± 254.62	578.43 ± 307.72
50	Alpha-pinene (M)	C80568	C10H16	909.2	419.79	1.21006	185.08 ± 40.16^cd^	119.70 ± 25.63^d^	258.78 ± 70.75^bc^	405.78 ± 60.52^a^	313.68 ± 33.08^b^
51	(E)-β-ocimene	C13877913	C10H16	1, 029.1	644.414	1.2656	83.19 ± 13.15^b^	72.30 ± 11.98^b^	74.79 ± 6.67^b^	420.05 ± 20.73^a^	73.04 ± 6.28^b^
52	Styrene	C100425	C8H8	881	375.364	1.41468	1,723.65 ± 232.59^a^	435.69 ± 130.12^b^	425.91 ± 64.50^b^	605.36 ± 41.94^b^	354.22 ± 141.50^b^
53	Diallyl sulfide	C592881	C6H10S	862.7	354.041	1.11647	375.31 ± 65.71^a^	75.68 ± 9.36^c^	214.94 ± 34.93^b^	266.66 ± 106.20^b^	56.41 ± 0.76^c^
54	Propylsulfide	C111477	C6H14S	891.1	387.109	1.14974	349.82 ± 30.77^c^	565.59 ± 5.73^b^	1,266.11 ± 26.28^a^	434.59 ± 43.40^bc^	570.64 ± 176.16^b^
55	2-Butoxyethanol	C111762	C6H14O2	896.4	396.148	1.21184	217.05 ± 44.65^c^	226.11 ± 58.50^c^	317.04 ± 45.64^bc^	375.60 ± 55.29^ab^	429.36 ± 63.73^a^
56	3-Butenenitrile	C109751	C4H5N	643.3	164.697	1.25904	307.53 ± 46.13^b^	492.47 ± 116.01^b^	1,340.36 ± 274.60^a^	1,240.22 ± 15.03^a^	407.97 ± 62.35^b^
57	*N*-nitrosodiethylamine	C55185	C4H10N2O	894	391.749	1.53126	179.82 ± 48.54^ab^	99.23 ± 11.83^b^	124.26 ± 20.65^b^	266.18 ± 61.57^a^	187.48 ± 67.70^ab^
58	2-Pentyl furan	C3777693	C9H14O	983.8	557.568	1.2612	137.25 ± 10.24^b^	138.10 ± 11.86^b^	151.58 ± 6.42^b^	557.24 ± 61.12^a^	160.59 ± 20.89^b^
59	2-Methyl-3-(methylthio) furan	C63012975	C6H8OS	946	487.809	1.15364	125.04 ± 14.21^c^	110.25 ± 20.14^c^	215.20 ± 27.37^a^	117.54 ± 3.15^c^	177.24 ± 26.79^b^

(M) and (D) denote monomer and dimer, respectively. Superscript a, b, c, and d in the same row denotes significantly different at *P* < 0.05.

Aldehydes were mainly generated from lipid oxidation and protein degradation, with the lower odor thresholds and having bigger influence on scallop flavor (19). Aldehydes changed most significantly in the fried scallops: compared with the control samples, the fried samples showed higher contents of heptenal, (E)-2-octenal, heptanal, hexanal, octanal, pentanal, 3-methylbutanal, and 3-methylthiopropanal (M). Besides, the boiled scallops contained much more contents of hexanal, pentanal and 3-methylbutanal (M) than the control samples. The roasted scallops contained more contents of pentanal, octanal (D) and 3-methylbutanal, while the microwaved scallops contained increased contents of pentanal, octanal (D), and hexanal. The identified aldehydes were mainly fatty aldehydes, generated from lipid oxidation and degradation. For example, hexanal (Fruit and leaf fragrances), heptanal (Nuts fragrance and green fragrance of fruit), octanal (Fruit and fatty odors) were also found in aquatic products like silver carps, tunas, and sturgeons (11, 20). Butanal gives green aroma and fruit fragrance and pentanal gives almond flavor (21, 22). In addition, (E)-2-octenal and heptenal derived from linoleic acid provide the fatty flavor (23). Branched aldehyde is generated from amino acid through Strecker degradation. For example, benzaldehyde commonly seen in meat products produces an almond-like sweet flavor, showing the smallest content in the boiled samples (21). 3-Methylthiopropanal conferred onion-like and meat aroma (22).

Ketones are mainly from lipid oxidation (19). The contents of most ketones in the fried samples were significantly higher than those in other samples. 2-Heptanone was thought to contribute to the meat aroma (24). 2-Pentanone, whose content was equally higher in the fried scallops than other samples, give food creamy and cheesy flavors (21). 2-Butanone increased significantly in the fried and microwaved scallops.

The main flavor substances in scallop muscles at the cooking stage were aldehydes and alcohols (6). However, unlike aldehydes, alcohols have higher odor thresholds, contributing smaller influence on flavor. 3-Methylthiopropanol, whose content was higher in raw and heated scallops, contributing a sweet onion flavor. 1-Octen-3-ol decreased after heating. It is a commonly seen flavor substance in aquatic products, known to contribute a peculiar smell of mushroom and mud, which decreases upon heating (25). 5-Methyl-2-furanmethanol, which increased significantly in the roasted, microwaved and fried scallops, provided a roast smell. 3-Methylbutan-1-ol gives an almond smell, with the content higher in the roasted scallops (22).

Esters provide sweet and fruit flavors; for example, ethyl hexanoate, ethyl acetate, and ethyl butyrate (26). Esters are esterified with the alcohols and free fatty acid generated from lipid oxidation (20). The content of isopropyl acetate increased after heating. Ethyl butyrate in the fried scallops was much more than that in other samples. Ethyl isovalerate and ethyl acetate decreased sharply after heating, probably because the content of volatile esters decreased as the heating temperature rises (17). There was only one kind of acid in scallops: propanoic acid, which increased after heating. Furans are important flavor substances in meat; they are generally generated from sugar degradation products, formed through Maillard reaction and usually have the meat and sweet flavor (19). The contents of 2-pentyl furan and 2-Methyl-3-(methylthio) furan were significantly higher in the fried and roasted scallops. Among them, 2-pentyl furan is commonly seen in meat as an important flavor substance and has fruit fragrance (27).

### Free amino acids contents in scallop samples

**FIGURE 2:**
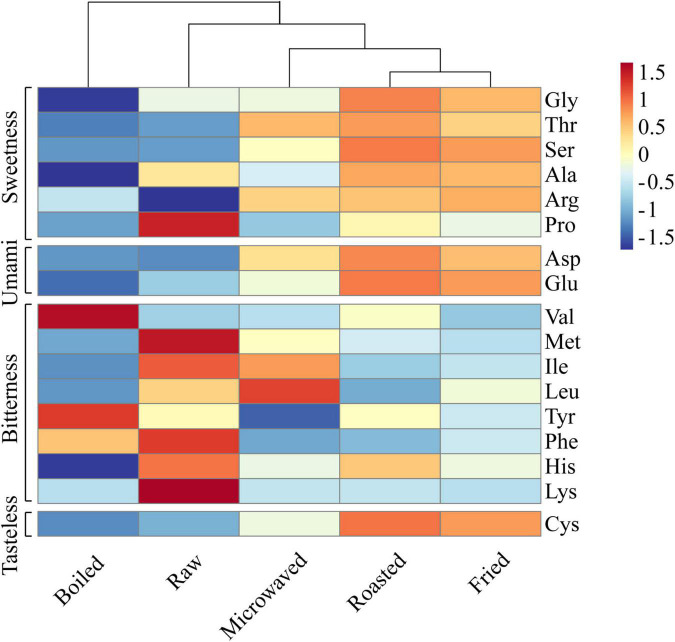
Heat map clustering of free amino acids in raw and cooked scallops.

Figure 2 was the heat map (the data were normalized with z-score) for free amino acids contents in scallops cooked by tested methods. The results showed that the roasted and fried scallops shared a higher level of similarity and can be clustered together. Except Pro, the roasted and fried scallops contained more sweet amino acids (Gly, Thr, Ser, Ala, and Arg), umami amino acids (Asp and Glu) and odorless amino acid (Cys) than other samples. In the microwaved scallops, sweet amino acids (Thr, Ser, and Arg) and umami amino acids (Asp and Glu) increased significantly than the control samples. Among them, Gly and Ala were the main sources of sweetness of shellfish (28). In terms of bitter amino acids, the red region representing high content was mainly concentrated in the control samples, while the blue region representing low content was mainly concentrated in the fried samples. Compared with other samples, the boiled scallops contained lower contents of most free amino acids, which was probably because the flavor compounds were dissolved in water during the boiling process (29). Heating duration and temperature differences also affected the protein degradation and caused further changes in amino acid contents (7). The heating medium in boiling was water; temperature of which was lower, while microwave heating lasted for a shorter time. Both heating methods feature less water loss, limiting the increase of amino acids (30). As the taste substances, free amino acids are crucial to food taste (31), with content changes contributing to the umami and sweet taste of scallop muscle.

### E-nose analysis of scallop samples

**FIGURE 3:**
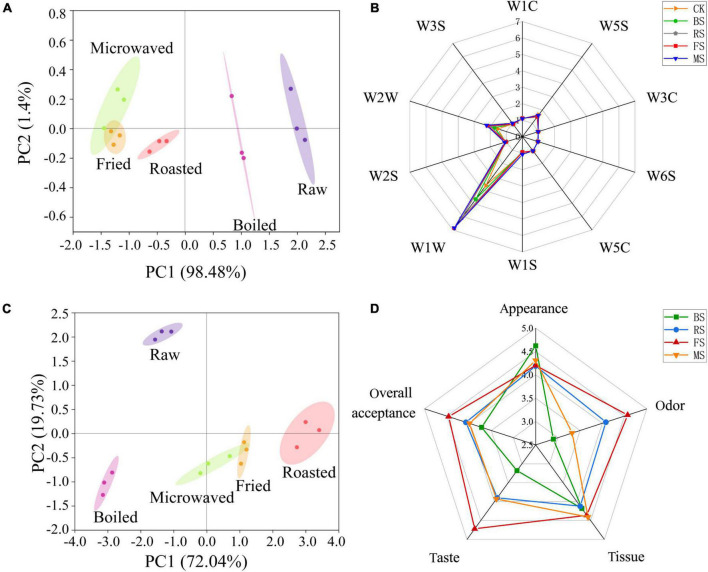
Electronic nose, electronic tongue, and sensory evaluation analysis of raw and cooked scallops. **(A)** Principal component analysis of electronic nose results. **(B)** Radar graph of electronic nose results. **(C)** Principal component analysis of electronic tongue results. **(D)** Radar graph of sensory evaluation results (CK, the control group; BS, boiled sample; RS, oven heated sample; FS, fried sample; MS, microwaved sample).

The principal component analysis method was used to develop the spatial distribution map, as shown in Figure 3A for different samples. PC1 and PC2 were 98.48 and 1.4%, respectively; the combined contribution rate was 99.88%, indicating that e-nose was able to differentiate well among the samples. The control and the boiled samples were distributed in the right half of the map, while the roasted, microwaved and fried samples were distributed in the left half. The control sample was far away from the samples cooked in the four different ways, indicating that all the four cooking methods caused changes to the smell of samples. The boiled sample was the closest to the control samples, followed by the roasted sample and then microwaved and fried samples. That means that the boiled sample had the smell closest to the control samples, while the fried and microwaved samples had the smell, which was the most different from the control samples. The radar map (Figure 3B) further supported the PCA results. Besides, their differences were mainly reflected in two metal receptors, including W1W (Sensitive to many terpenes and organic sulfur compounds) and W2W (Sensitive to aromatic compounds and organic sulfide); the fried and microwaved sample were the most responsive and the control sample was the least one. This was consistent with the HS-GC-MS results, where 3-methylthiopropanal (an organic sulfide) contributed most to the signal changes of W1W and W2W metal receptors (Figure 3B).

### E-tongue analysis of scallop samples

The principal component analysis method was used to develop the spatial distribution map (Figure 3C) of different samples. PC1 and PC2 were 72.04 and 19.73%, respectively, and the combined contribution rate was 91.77%, indicating that e-tongue was able to differentiate well among the samples. The control sample was located in the second quadrant, far away from the samples cooked in four different ways, indicating all the four cooking methods caused changes to the taste of the scallop muscle. Only the fried and microwaved samples overlapped, indicating that the two samples had similar taste, while other samples differed greatly in taste.

### Sensory evaluation of scallop samples

The radar map for the sensory assessment of scallop muscle cooked by different ways is shown in Figure 3D. The samples differed little in appearance among samples but differed greatly in smell and taste. In terms of smell, the fried and roasted samples had higher sensory scores. In terms of taste, the fried, roasted and microwaved samples had higher scores. Overall, the fried sample was more receptive than other samples, while the boiled sample was least receptive. The sensory scores for smell and taste varied greatly among samples, affecting the overall receptivity to a greater degree. Interestingly, associated with the results of the e-nose- and e-tongue-principal component analysis, boiled samples were highly similar to raw samples in taste and volatile odor substances. On the one hand, this high degree of similarity seems to better achieve the goal of maintaining the original sensory qualities of fresh scallops. However, it should not be ignored that the boiled samples obtained the lowest sensory score among the cooked samples. Therefore, this way of cooking is not the best way to meet the needs of consumers.

### The analysis of microstructures and texture profiles in scallop samples

The TPA method was used to simulate the secondary chewing process in human oral cavity and analyze the influence of different cooking methods on the taste of scallop muscle. The roasted sample was much harder and chewier than the other three samples; also, it was much more elastic than the boiled sample (Table 2).

**TABLE 2:** Changes in the texture of heating scallops.

Groups	Hardness (N)	Springiness (mJ)	Chewiness (mJ)
Boiled	2.77 ± 0.31^b^	1.71 ± 0.09^b^	2.25 ± 0.13^b^
Roasted	4.03 ± 0.67^a^	2.34 ± 0.10^a^	4.01 ± 1.08^a^
Fried	3.00 ± 0.60^b^	2.02 ± 0.23^ab^	2.46 ± 0.48^b^
Microwaved	2.93 ± 0.15^b^	1.91 ± 0.45^ab^	2.35 ± 0.14^b^

Superscript a, b, and c in the same column denotes significantly different at *P* < 0.05.

**FIGURE 4:**
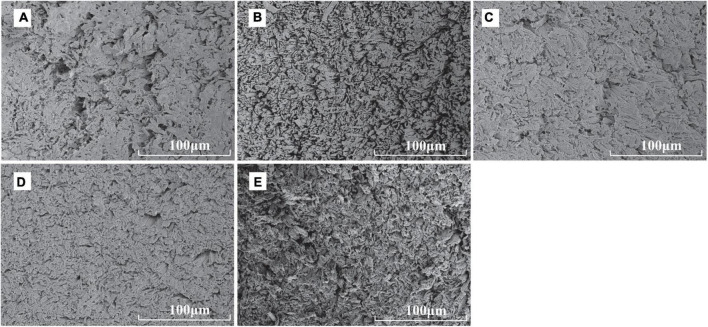
Scanning electron micrographs of raw and cooked scallops (500× magnification, **(A)** CK, the control group; **(B)** BS, boiled sample; **(C)**: RS, oven-heated sample; **(D)** FS, fried sample; **(E)** MS, microwaved sample).

We used the scanning electron microscopy to observe the microstructure of the section of scallop muscle cooked in different ways. The muscle fibers in the control sample were arranged most loosely, while the seams among muscle bundles notably shrank after heating during cooking by different methods, probably caused by the loss of water during heating (Figure 4). The muscle fibers in the boiled and microwaved samples were arranged relatively loose, while the muscle fibers in the fried and roasted samples were tightly arranged with smaller seams. Such difference in muscle fiber arrangement might be the reason behind the difference in hardness, elasticity and chewiness values (32). Besides, the muscle fibers of microwaved sample were arranged in the most disorder manner, probably related to the principle of microwave heating, ultimately improving the tenderness of scallop muscle (15). Unlike frying, boiling or roasting, where the transmission of heat takes place through conduction from outside to inside of the material being heated, microwave system heats food through water molecular friction (30), which involves fast high-frequency vibrations and molecular polarization (33).

### Correlation analysis of the data

Taste, volatile, and texture are important factors affecting the sensory assessment of foods. One physicochemical indicator cannot reflect the cross-effect of these factors on sensory (34). So, we used correlation analysis to further explore the effect.

**FIGURE 5:**
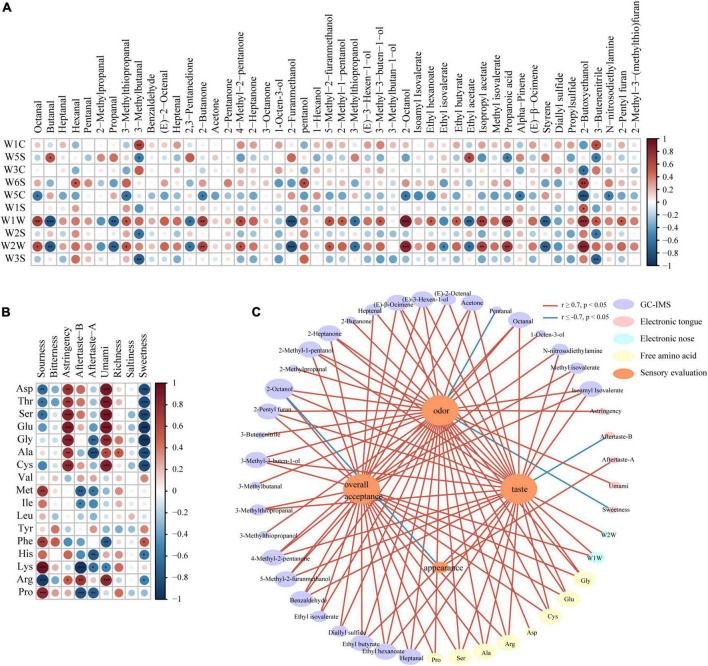
Correlation analysis: **(A)** Between volatile compounds with e-nose results and **(B)** between free amino acids with e-tongue results (*0.01 < *P* < 0.05, **0.001 < *P* < 0.01; ***P < 0.001). **(C)** Correlation networks between volatile compounds, e-nose results, free amino acids, e-tongue results with sensory scores, based on Pearson correlation coefficients (| *r*| > 0.7, *P* < 0.05) (Red represents positive correlations, and blue represents negative correlations).

The results of the e-nose and the correlation heat map for volatile organic compounds identified through HS-GC-IMS (Figure 5A; *P* < 0.05) showed the correlation between the e-nose metal receptor signals and the concentration of flavor substances. Significant correlation appeared in W1W and W2W metal receptors; W1W metal receptor (Sensitive to many terpene and organic sulfur compounds) showed a significant positive correlation with octanal, 3-methylthiopropanal, 2-butanone, 4-methyl-2-pentanone, 5-methyl-2-furanmethanol, 2-methyl-1-pentanol, 3-methyl-3-buten-1-ol, 2-octanol, ethyl hexanoate, ethyl butyrate, isopropyl acetate, propanoic acid, 2-butoxyethanol, 3-butenenitrile, and 2-pentyl furan, while W2W metal receptor (Sensitive to aromatic compounds and organic sulfide) was significant positively correlated with octanal, 3-methylthiopropanal, 2-butanone, 4-methyl-2-pentanone, 5-methyl-2-furanmethanol, 2-octanol, isopropyl acetate, propanoic acid, and 2-butoxyethanol. Such compounds affected the e-nose results to a larger degree.

Many researches showed that free amino acids (FAAs) were positively correlated to taste (35). According to the e-tongue results and the heat map for the contents of free amino acids (Figure 5B; *P* < 0.05), the contents of Asp, Thr, Ser, Glu, Gly, Ala, Cys, and Arg had significant positive correlation with the umami signal and convergence signal of e-tongue, and was negatively correlated to the sweet signal of e-tongue. The acid signal of e-tongue was negatively correlated with Asp, Thr, Ser, and Arg, while positively correlated with Met, Phe, Lys, and Pro. The aftertaste signal of e-tongue was negatively correlated with Gly, Ala, Met, Ile, His, Lys, and Pro, while positively correlated with Arg. Besides, Phe and Lys were negatively correlated with the umami signal; Arg was positively correlated with the umami signal; His and Arg were negatively correlated with the sweet signal; Phe was positively correlated with the sweet signal; all the 17 free amino acids had no significant correlation with the bitter and salty signals of e-tongue.

Glu and Asp were umami amino acids; both of them showed a significant positive correlation (*P* < 0.001) with the umami signal of e-tongue. Glu was positively correlated with the sensory score of taste and overall sensory score (| *r*| ≥ 0.7, *P* < 0.05). Besides, as shown in Figure 5B, Arg was positively correlated with the umami signal, while Phe was positively correlated with the acid signal, which was consistent with previous results (0.001 < *P* < 0.01) (28, 31).

Figure 5C is the network diagram showing how sensory scores are correlated to volatile organic compounds, free amino acids contents, e-nose results and e-tongue results (|*r|* ≥ 0.7, *P* < 0.05). Among the 48 volatile flavor compounds separated through HS-GC-IMS, 30 compounds were correlated with sensory scores. Among them, 23 flavor compounds, including benzaldehyde, heptanal, and 2-pentyl furan, were positively correlated with the sensory score of odor; pentanal was negatively correlated with the sensory score of odor; 23 flavor compounds, including octanal, and heptanal were positively correlated with the sensory score of taste; 23 flavor compounds, including octanal, and benzaldehyde were positively correlated with overall sensory scores. Eight of the 17 free amino acids were significantly correlated with the sensory scores. The contents of Ala, Glu, Arg, Ser, Gly, and Cys were positively correlated with taste, odor and overall sensory scores. Besides, the content of Pro and Asp were positively correlated with the sensory score of odor, while the content of Pro was positively correlated with the overall sensory score. E-nose sensor W1W was positively correlated with the overall sensory score. Only the aftertaste-B signal of e-tongue was negatively correlated with the sensory score of taste. As the sensory score of structure had no significant correlation with other indicators, it was not indicated in the figure.

Among the free amino acids that were positively correlated with the sensory score of taste and the overall sensory score, all the free amino acids had sweet or umami taste except Cys (which is tasteless free amino acid) (| *r*| ≥ 0.7, *P* < 0.05). However, sweet free amino acids, including Gly, Thr, Ser, and Ala were negatively correlated with the sweet results of e-tongue (*P* < 0.001), probably related to the limitations of e-tongue. Shen et al. (36) pointed out that the electric potential sensor of e-tongue might absorb compounds, leading to differences between the response value of the sensor and sensory scores. Like all the analytical systems, e-tongue can’t fully replace human senses, because human eating involves chewing and is a process featuring dynamic sensing of taste. However, e-tongue can only measure liquid samples under static condition, which cannot simulate a complete eating process (13).

Interestingly, some researches showed 3-methylthiopropanal was able to positively activate human T1R1/T1R3, the taste receptor of umami (37). In this research, the content of 3-methylthiopropanal increased in the fried and microwaved samples, which was positively correlated with the taste sensory score. Besides, many volatile organic compounds separated through HS-GC-IMS had important positive role in the taste, smell and overall sensory scores. That indicated that rich flavor compounds not only offered the tasters a better smell experience, but also affected taste feelings. FAAs can make the precursor of flavor substances, changing food flavors together with volatile organic compounds (38). The interactions between senses of smell and taste were also seen in the results of Merlo et al. (39).

## Conclusion

This research provided a comprehensive method that uses HS-GC-IMS, e-nose, e-tongue, sensory analysis, and free amino acid tests to identify the sensory characteristics of scallop muscle cooked in different ways. It also explained the differences of taste among samples through texture profile and microstructure analysis. Overall results showed that fried scallop had the best sensory score, probably because of the significant increase in aldehydes and ketones, caused by a higher degree of lipid oxidation and Maillard reaction as well as the increase in furans, umami, and sweet free amino acids. Boiled scallop had the lowest sensory score, probably related to the decrease in sweet and umami amino acids, caused by the loss of water-soluble compounds and fewer contents of volatile organic compounds. The interactions between senses of smell and taste resulted from volatile organic compounds and free amino acids were crucial to the formation of the sensory quality of scallop.

## Data availability statement

The original contributions presented in this study are included in the article/Supplementary material, further inquiries can be directed to the corresponding authors.

## Author contributions

YW and JG conceived and designed the study. YW performed the experiments, analyzed the data, and wrote the manuscript. ZW and KM performed the statistical analysis. GT, JG, BC, and XB contributed to revisions of the manuscript. YS, JG, XB, and JL projected administration. All authors have read and approved the manuscript.
