# Supplementary material for: Effects of four cooking methods on flavor and sensory characteristics of scallop muscle
Source: Front Nutr. 2022 Oct 12;9:1022156. doi: 10.3389/fnut.2022.1022156 (PMC9605777; doi:10.3389/fnut.2022.1022156)
Supplement: Supplementary file 1 [file Data_Sheet_1.docx]

Supplementary materials：

**Table 1**

Free amino acids of raw and heating scallops (mg/100g) .

| Components | Raw | Boiled | Roasted | Fried | Microwaved |
| --- | --- | --- | --- | --- | --- |
| Gly | 167.47±4.08^c^ | 128.97±0.90^d^ | 197.83±3.40^a^ | 189.20±2.26^b^ | 168.87±8.08^c^ |
| Thr | 11.10±0.17^b^ | 9.77±0.32^b^ | 24.83±3.15^a^ | 22.17±1.29^a^ | 23.63±1.01^a^ |
| Ser | 4.50±0.26^c^ | 4.20±0.17^c^ | 21.33±0.72^a^ | 20.03±1.33^a^ | 13.50±4.29^b^ |
| Ala | 51.40±9.93^ab^ | 25.40±0.26^c^ | 57.60±1.28^a^ | 55.90±1.75^a^ | 43.00±5.58^b^ |
| Val | 85.43±7.43 | 99.63±9.39 | 89.80±15.47 | 85.20±10.86 | 86.43±8.35 |
| Lys | 55.87±10.81^a^ | 10.83±2.70^b^ | 12.43±3.91^b^ | 11.17±0.51^b^ | 12.17±1.79^b^ |
| Arg | 10.73±0.21^c^ | 60.43±4.49^b^ | 104.43±7.55^a^ | 110.77±12.53^a^ | 99.43±3.23^a^ |
| Pro | 66.70±0.20^a^ | 18.73±6.86^c^ | 40.6±0.20^b^ | 35.13±7.10^b^ | 24.03±0.57^c^ |
| Asp | 11.47±0.06^c^ | 12.27±0.85^c^ | 33.77±3.16^a^ | 30.37±0.06^ab^ | 27.93±6.02^b^ |
| Glu | 40.77±1.00^c^ | 24.07±1.92^d^ | 88.23±3.41^a^ | 83.00±1.92^a^ | 57.80±8.64^b^ |
| Met | 184.17±17.33^a^ | 130.87±10.51^b^ | 143.80±12.04^b^ | 140.00±23.35^b^ | 152.33±9.33^b^ |
| Ile | 170.77±24.09 | 126.50±25.96 | 134.43±30.33 | 138.70±17.41 | 164.23±20.28 |
| Leu | 107.23±16.97^ab^ | 67.97±0.55^c^ | 71.40±14.82^c^ | 92.80±18.38^bc^ | 127.97±10.79^a^ |
| Tyr | 54.53±9.35^ab^ | 61.60±10.76^a^ | 54.10±2.14^ab^ | 51.50±5.67^ab^ | 45.87±3.66^b^ |
| Phe | 36.83±11.45^a^ | 30.80±1.15^ab^ | 19.43±0.99^c^ | 22.87±0.06^bc^ | 18.30±0.26^c^ |
| His | 10.33±0.21^a^ | 4.53±0.21^b^ | 9.30±2.19^a^ | 7.77±0.42^a^ | 7.67±2.14^a^ |
| Cys | 12.00±1.47^bc^ | 10.53±1.12^c^ | 24.07±1.23^a^ | 22.80±3.51^a^ | 16.87±5.02^b^ |
| Total | 1081.30±56.31^a^ | 827.10±18.86^b^ | 1127.40±70.08^a^ | 1119.37±64.96^a^ | 1090.03±37.14^a^ |

Superscript a, b, c, and d in the same row denotes significantly different at *P* < 0.05.

**Table 2**

Sensory evaluation criteria

| Evaluation index | Standard declaration | Score |
| --- | --- | --- |
| Appearance | The surface is smooth, and the color is full and uniform. | 3.0-5.0 |
|  | Rough shape, smooth surface, uneven color. | 1.6-2.9 |
|  | Extremely rough in shape and uneven in color. | 0-1.5 |
| Odor | Rich meaty aroma, umami and other pleasant smell, a little fishy odor, no peculiar smell. | 3.0-5.0 |
|  | Fishy odor, a little meat aroma, umami and other pleasant smell, no peculiar smell. | 1.6-2.9 |
|  | Rich fishy odor and peculiar odor, almost no meat, umami and other pleasant smell . | 0-1.5 |
| Tissue | Compact structure, delicate meat quality, moderate chewing resilience, no cracking separation. | 3.0-5.0 |
|  | Loose structure, general chewing resilience, less cracking and separation of soft collapse of the phenomenon of molding. | 1.6-2.9 |
|  | Loose structure, shellfish split or separated, skin bubbles, separated from the interior, soft collapse is not formed, chewing resilience is too large or too small. | 0-1.5 |
| Taste | Umami and sweet taste, no bitter greasy acerbity and other unpleasant taste. | 3.0-5.0 |
|  | A little sweet and umami taste, no unpleasant taste. | 1.6-2.9 |
|  | Almost no sweet and umami taste, but obviously unpleasant taste. | 0-1.5 |
